# Tuberculosis with cavities? Rapid diagnosis of *Rhodococcus equi* pulmonary infection with cavities by acid-fast staining: A case report

**DOI:** 10.3389/fpubh.2022.982917

**Published:** 2022-09-16

**Authors:** Yuhang Jiang, Jian Li, Weichao Qin, Yuan Gao, Xin Liao, Yan Zeng

**Affiliations:** ^1^Department of Laboratory Medicine, Chongqing University Jiangjin Hospital, Chongqing, China; ^2^Department of Laboratory Medicine, Central Hospital of Jiangjin District, Chongqing, China; ^3^Department of Respiratory Medicine, Chongqing University Jiangjin Hospital, Chongqing, China; ^4^Department of Laboratory Medicine, Jiangjin District Maternity and Child Health Hospital, Chongqing, China

**Keywords:** *Rhodococcus equi*, acid-fast staining, human immunodeficiency virus, matrix-assisted laser desorption/ionization time-of-flight mass spectrometry, pulmonary infection, case report

## Abstract

*Rhodococcus equi* is a conditionally pathogenic bacterium widely distributed in soil, water, and marine environments, which can cause respiratory infections, pleurisy, blood and even bone marrow infections in immunocompromised people, and particularly in patients with acquired immunodeficiency syndrome (AIDS). This case report describes a patient with initially suspicion of tuberculosis (TB) as an outpatient in a TB clinic. However, laboratory findings identified *R. equi* in his sputum sample based on a positive acid-fast stain, which was highly suggestive of a pulmonary infection caused by *R. equi*. The patient was subsequently admitted to the respiratory unit for treatment. Once the source of infection was identified, the patient was treated with a combination of antibiotics for 2 weeks and was discharged with a significant improvement in symptoms.

## Introduction

*Rhodococcus equi* (hereafter *R. equi*), belonging to the genus Rhodococcus of the family Nocardiaceae, is named because it causes pulmonary infection in foals (1). *Rhodococcus equi* is a Gram-positive aerobic bacteria that produce fungus-like mycelia, which detach into rods and/or spheres within a short period of time. The bacteria were previously classified as Corynebacterium (2). The appearance of rod and/or globular forms correlates with the stage of culture, the type of specimen, and the culture conditions. The clinical pathogenicity of *R. equi* has been linked to human immunodeficiency virus (HIV) infection, and reports of AIDS co-infection with *R. equi* are increasing. Nonetheless, there are few detailed reports describing laboratory identification processes. Therefore, this report provide details on the laboratory identification (including molecular biology) and morphological characteristics of *R. equi*.

## Case report

The patient was a 35-year-old male, white-collar worker, with no contact with livestock. He reported cough with sputum and hemoptysis after a cold 3 months prior to consultation, but without fever, chills, hot flushes, or night sweats. As these symptoms did not attract the patient's attention, the patient did not seek medical attention at a hospital for appropriate treatment. Approximately a month prior to the patient's first visit, the patient developed recurrent fever with chills and night sweats, with a maximum temperature of 38.9°C occurring mainly at night. However, the patient still did not seek medical attention. He recently developed new symptoms, such as weakness, lack of appetite, and poor mental health, and was seen at another hospital.

## Clinical findings

**Figure 1:**
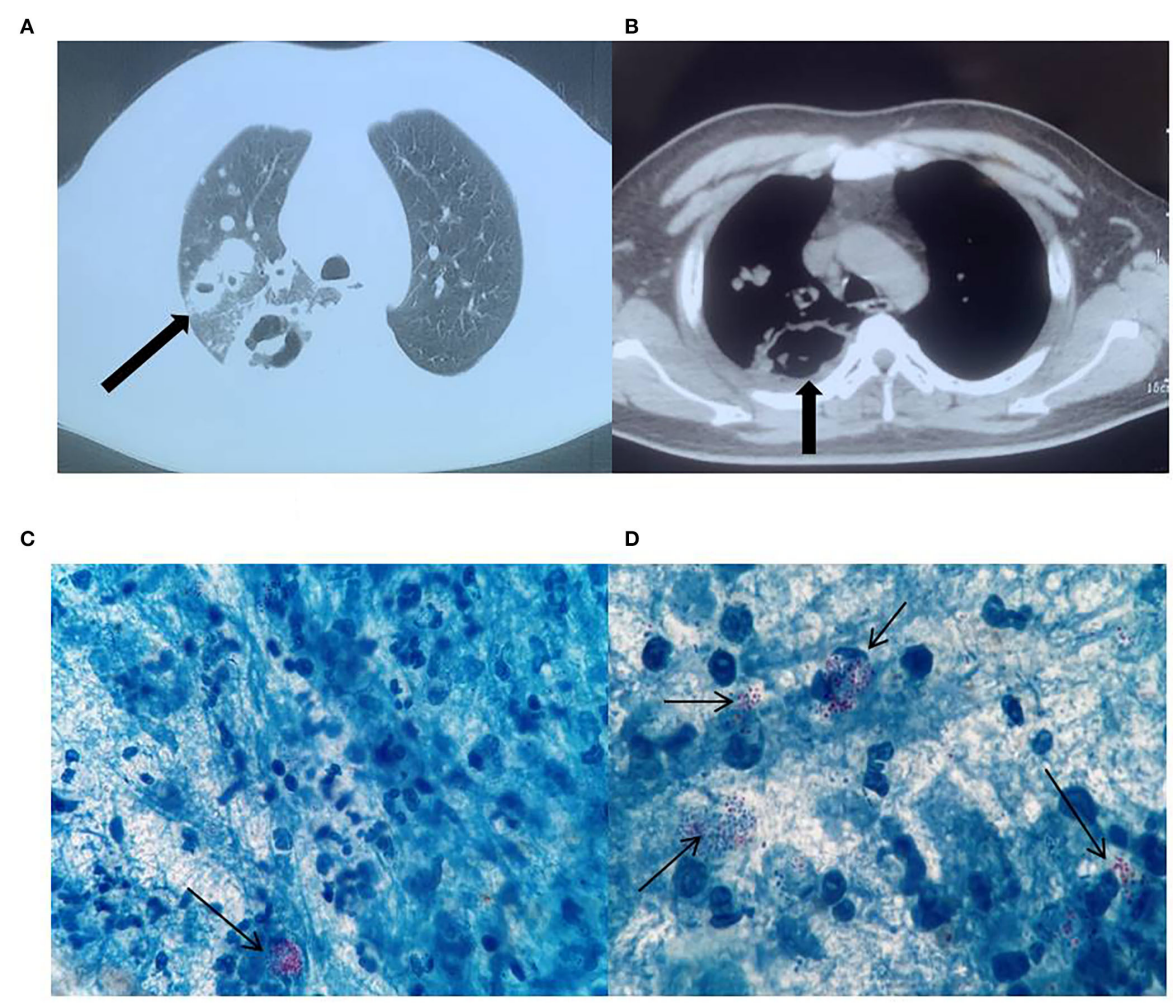
Chest computed tomography (CT) images. Right lung cavity **(A)**, thickening of and adherence to the right pleura **(B)**. Acid-fast bacteria in the microscopic field of view **(C,D)**, ×1000.

Physical examination of the patient on admission revealed a body temperature of 38.9°C, pulse rate of 86 beats/min, respirations of 20 breaths/min, and blood pressure of 128/78 mmHg. The patient has bilateral thickened bilateral lungs and obstructed breath sounds, coarse wet rales were heard in the right lung, and no pathological murmurs were heard at rest. The findings of a chest computed tomography (CT) (performed at another hospital) suggested possible secondary tuberculosis (TB) with cavitation in the right upper lung and middle lung lobes and thickened adhesions in the right pleura (Figures 1A,B). The patient was directed to the TB clinic to confirm the diagnosis of pulmonary TB where three sputum samples were obtained. Our laboratory performed acid-fast staining of these sputum samples and a large number of acid-fast bacteria, which could be visible under the microscope (Figures 1C,D). Nonetheless, the microscopic morphology was globular and partially colored clusters, which was not typical of that common *Mycobacterium tuberculosis*, thus the patient was suspected of infecting with *R. equi*. We then alerted the clinician about the findings, and the patient was admitted to the respiratory unit of our hospital for systematic treatment.

## Laboratory examinations

The inflammatory indicators for this patient during treatment and during the follow-up review after discharge are presented in Table 1. Furthermore, the patient's HIV antibody screening test was positive and the results of the CD4+ T lymphocyte count were 31 cells/μl, the two findings that were highly suggestive of HIV infection. Furthermore, the patient's T-cell spot test was negative for TB infection, which helped to exclude pulmonary TB. More importantly, the culture of the sputum sample revealed colonies of *R. equi* (semiquantitative: 4+), which provided strong evidence of pulmonary infection with *R. equi*.

**Table 1:** Indicator of inflammation in this patient.

	**Day 1**	**Day 4**	**Day 7**	**Day 13**	**D-week 2**	**D-week 8**
WBC count (×10^9^/L)	17.60	18.09	11.59	6.00	3.30	4.21
CRP (mg/L)	>200.00	172.09	143.08	39.35	2.29	5.26
PCT (ng/mL)	0.88		1.34	<0.50		
IL-6 (pg/mL)	49.52		15.77	<7.00		

WBC, white blood cell; CRP, C-reactive protein; PCT, procalcitonin; IL-6, interleukin 6.

D-week, the number of weeks since the patient was discharged; a blank indicates that the item was not tested at that time.

## Bacterial identification

**Figure 2:**
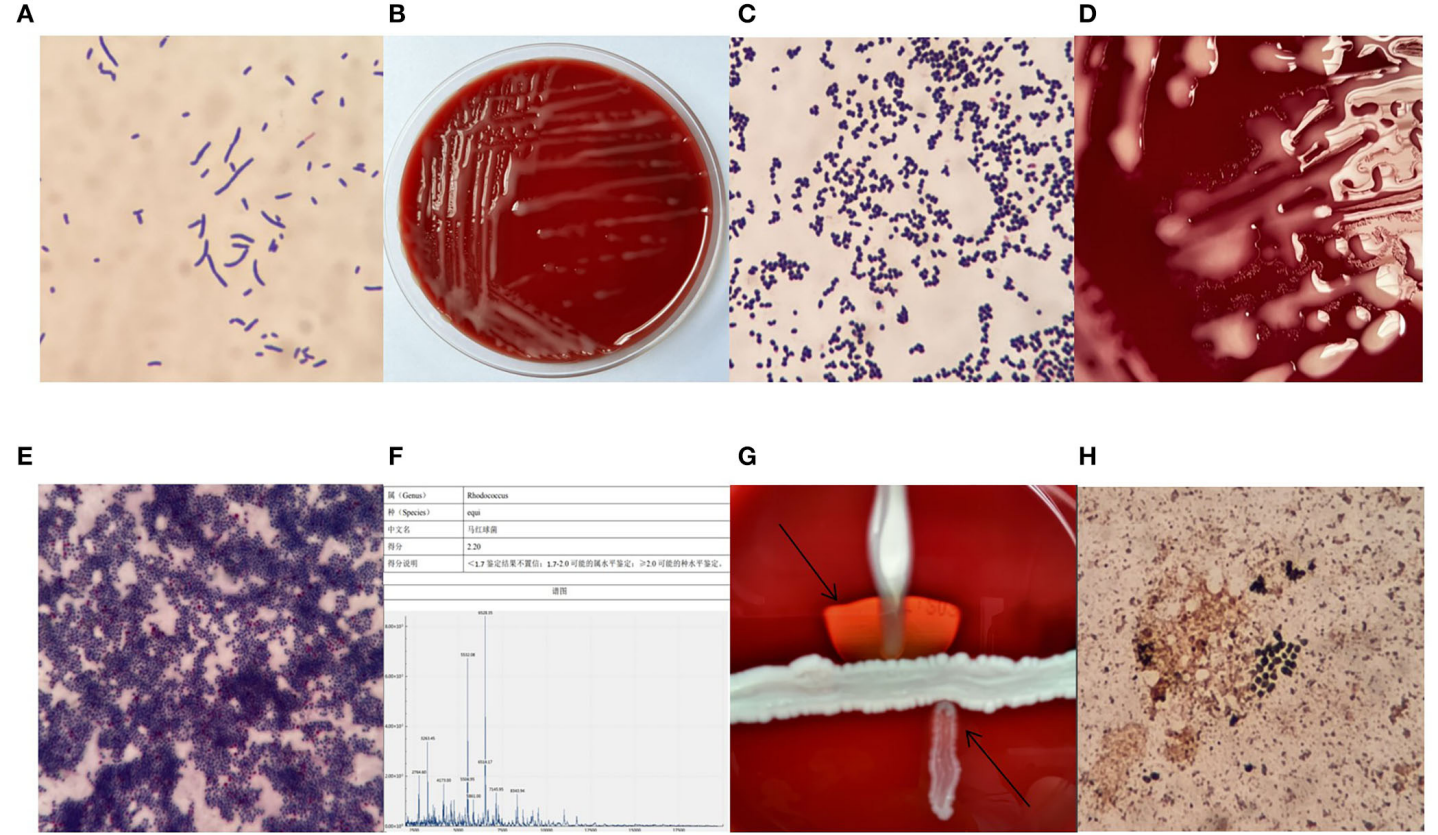
Gram stain after 6 h of pure culture **(A)** ×1,000, colony morphology after 24 h of pure culture **(B)**, Gram-stained morphology after 24 h of pure culture **(C)** ×1,000, fungal pseudomycorrhizal-like material **(D)**, the morphology of weak acid-fast stain **(E)** ×1,000, mass spectrometer identification result **(F)**, the Christie–Atkins–Munch–Peterson (CAMP) test **(G)**, the morphology of hexamine silver staining **(H)** ×1,000.

Patient's sputum samples were spread on a Colombian blood agar plate, a Chocolate agar plate, an MacConkey agar plate, and an Sabouraud agar plate (all plates were produced in China by Zhengzhou Antu Biological Bioengineering Co.). After 24 h of incubation at 35°C and 5% CO_2_, only on the Columbia blood agar plate grew a large number of mucinous colonies and we selected the mucinous colonies for pure culture, after 6 h of pure culture, colonies from the original area of the plate were selected for Gram staining, and Gram-positive rods and mycelium-like fungal bodies were visible under the microscope (Figure 2A). After 24 h of pure culture, mucinous colonies with a smooth, raised, light orange surface and ~1 mm in size, without hemolysis, developed on the blood agar plate (Figure 2B), and the Gram staining pattern of colonies at this stage was globular in clusters under the microscope (Figure 2C). After 48 h of culture, an aerial mycelium-like material formed around the colonies (Figure 2D). The colonies were stained with a weak acid-fast stain after 48 h of pure culture, and some bacteria were observed to resist acid staining under the microscope (Figure 2E). This bacterial strain was identified with the French Mérieux VITEK-2 Compact GP identification card (complete blood count (CBC) identification cards are not routinely purchased in our laboratory) as a low-confidence *Kocuria spp*. Next, we evaluated the strain by mass spectrometry using the Zhongyuan Huiji Micro Typer MALDI-TOF and identified it as *R. equi* (Figure 2F). We extracted deoxyribonucleic acid (DNA) fragments from pure colonies and amplified 16S ribosomal ribonucleic acid (RNA) using the primers 7F and 1540R. The purified polymerase chain reaction (PCR) products were used for sequence comparison using BLAST in the National Center for Biotechnology Information database website, and a fragment of 1,433 bp in length was selected for comparison and determined to correspond to the *R. equi* strain (ATCC 6939) with a similarity of 99.86%. *The R. equi* catalase test and urease were positive and did not decompose glucose. The results of the Christie–Atkins–Munch–Peterson (CAMP) test were observed after 24 h incubation on a blood agar plate with *Staphylococcus aureus* (ATCC 25923) and *Listeria monocytogene* as controls. The results showed that *R. equi* was hemolyzed in the form of an arrowhead near *S. aureus*, while *L. monocytogene* was hemolyzed in the form of a match head near *S. aureus* (Figure 2G). Furthermore, the sputum sample was also stained with silver hexamine to determine whether the patient was infected with *Pneumocystis carinii*, but dark black *R. equi* colonies were observed by microscopy (Figure 2H), which confirmed that silver hexamine staining could also stain acid-resistant bacteria the same dark brown color as fungi.

## Susceptibility to antibiotics

We prepared bacterial suspensions with a standard turbidity of 0.5 McFarland and tested the susceptibility of the strains to antibiotics by the *E*-test method; the results are presented in Table 2.

**Table 2:** Antibiotic susceptibility test for *Rhodococcus equi*.

**Antibiotics**	**MIC (mg/L)**	**Antibiotics**	**MIC (mg/L)**
Rifampin	1	Imipenem	1
Levofloxacin	1	Penicillin	8
Vancomycin	0.25	Clindamycin	4
Erythromycin	0.5	Sulfamethoxazole	2
Linezolid	1	Daptomycin	4

MIC, minimum inhibitory concentration.

## Clinical treatment

After the pathogen was identified, the clinician immediately treated the patient with a three-drug combination of levofloxacin combined with rifampicin and meropenem (the patient started treatment with the combination of drugs on day 3). After a week of combined treatment, the patient's symptoms and inflammatory index began to improve, and after 2 weeks the patient was discharged with a significant improvement in symptoms. The patient's condition did not deteriorate at the 2-week follow-up. The patient was asked to continue taking oral antibiotics until the CD4^+^ T lymphoid count reached 200 cells/μl after antiviral treatment, and regular monthly follow-up of inflammatory indicators and chest CT.

## Discussion

*Rhodococcus equi* belongs to the genus Rhodococcus. Currently, there are 57 species belonging to the genus Rhodococcus, the most common and relevant to clinical medicine being *R. equi, R. roseus*, and *R. rhodochrous*. *R. equi* is widely distributed in all living environments (except Antarctica) and is a conditionally pathogenic bacterium (1). *Rhodococcus equi* is closely associated with HIV infection and approximately two-thirds of patients infected with *R. equi* are also infected with HIV (3), which should alert clinicians whether the infection with *R. equi* is accompanied by other factors that contribute to the patient's immune deficiency, such as HIV infection. Infections caused by *R. equi* are prone to misdiagnosis or underdiagnosis. One of the reasons for this is misinformation provided to clinicians by testing laboratories. The Gram staining pattern of *R. equi* is related to the stage of culture, and when a bloodstream infection occurs, Gram-positive, rod-shaped bacilli may be found in blood specimens, although the blood culture may only be positive for a short period of time. Reporting misinformation to clinicians may result in the judgment of contaminating bacteria and thus overlooking the correct treatment plan. Tuon et al. described two deaths as a result of an outbreak of *R. equi* infections, both incorrectly identified as *Corynebacterium spp*. This misinformation led clinicians to interpret the findings as contaminating bacteria despite multiple positive blood cultures (4). Laboratories using the Mérieux VITEK-2 Compact should be aware that *R. equi* is listed in the identification catalog on the CBC identification card; whereas most laboratories using this instrument are equipped with the GP card (Gram-Positive identification card). However, neither the GP identification card nor the anaerobic and corynebacteria identification card (ANC) identification card can accurately identify *R. equi* and will give incorrect results. If laboratories provide misinformation to clinicians, this will also result in patients not receiving the correct treatment and timing. Thus, mass spectrometry is considered particularly suitable for the rapid and accurate identification of bacterial species, although specific staining techniques can also assist in the differential diagnosis of infections caused by *R. equi*, as in this case, where we initially identified a suspected infection of *R. equi* using acid-fast staining. However, the clinician's lack of experience and awareness of *R. equi* infection may also lead to misdiagnosis or underdiagnosis. As the foci of lung infection by *R. equi* are usually diffuse, pleomorphic, and associated with cavitation of the lung, they can easily be misdiagnosed as pulmonary TB with cavitation (5–7). The patient in this case had the same imaging characteristics and was also initially highly suspected of having pulmonary TB. Although both infections exhibit cavitation, the cavities in tuberculous caseous pneumonia are worm-eaten-like hollows, whereas the cavities in *R. equi* are formed when *R. equi* produces continuous lesions in the alveolar wall, and the cavities tend to have compartmentalized changes. Furthermore, pulmonary *R. equi* infections can be divided into exudative, solid, and abscess-forming stages. Early lesions are often detected by a small patch of slightly high-density shadows, but if not treated effectively, they can develop into solid lesions, forming nodular or mass-like lesions, which are remarkably similar to malignant tumors and are often combined with enlarged mediastinal lymph nodes. *Rhodococcus equi* pneumonia can show parenchymal changes in the spherical lung, some of which are well defined, but most have blurred margins and are broadly conical with the base located on the pleural side. In contrast, inflammatory lesions surrounding a lung tumor are mostly characterized by an obstructive inflammation distributed distally to the mass. The exudative inflammation surrounding *R. equi* pneumonia has no clear pattern, and there are often exudative inflammatory lesions from other lobes.

Treatment of *R. equi* infections generally recommends a combination of antibiotics (8). The Sanford Guide to Antimicrobial Therapy recommends azithromycin, levofloxacin, or rifampicin as the first choice for the anti-infective treatment of *R. equi*; vancomycin or imipenem plus levofloxacin or azithromycin or rifampicin as the second choice. Penicillin, cephalosporins, clindamycin, tetracycline, and cotrimoxazole should be avoided. It is important to note that although vancomycin may be susceptible to *R. equi in vitro*, because *R. equi* is a facultative intracellular parasitic bacterium, vancomycin cannot achieve expected intracellular concentrations, leading to reduced activity against intracellular *R. equi* and failure of treatment. Therefore, clinicians should choose antibiotics that have good cell penetration to treat the infection. The treatment period for *R. equi* infections is long and requires prolonged medication, and some patients continue to require oral medications after discharge from the hospital. The treatment period varies from a few months to a few years, while cavitary lesions should be treated for longer periods of time, as there is also a risk of recurrence (1, 8, 9). Capdevila et al. reported that only half of the 78 patients infected with *R. equi* in their hospital were cured after prolonged antibiotic treatment, it is easy to relapse after treatment, and the probability of recurrence is close to 50% (6).

Furthermore, early use of highly active antiretroviral therapy once HIV infection is diagnosed may improve the prognosis of HIV coinfection with *R. equi* and increases the chances of recovery; in untreated HIV-infected individuals, the relative risk of death from *R. equi* infection was 53 times higher than in those who received appropriate antiretroviral therapy (10). Many cases of treatment failure are due to the lack of effective antiretroviral therapy in combination with treatment for *R. equi* (11–13). For HIV patients with *R. equi* infection, it is not only necessary to monitor their inflammatory indicators [such as white blood cell (WBC) count and C-reactive protein (CRP)] at a later stage, but also the CD4+ T lymphocyte count should be monitored. Currently, there is no standard reference for the specific values of these parameters. When the CD4+ T lymphocyte count returns to more than 200 cells/μl after receiving antiretroviral therapy, this could indicate that the patient has achieved some immunity, and the treatment can be adjusted according to the patient's specific clinical condition.

It should be noted that, in this case, we initially identified a large number of acid-fast cocci in his sputum through acid-fast staining, and when combined with our experience, we correctly determined that it was *R. equi* and promptly informed the clinician and patient in advance so that they could prepare a treatment plan in advance, which saved the patient and the doctor considerable time and effort and made our findings more clinically valuable.

In conclusion, although the number of reports of *R. equi* is gradually increasing, there are still many laboratories that have difficulty in identifying the bacteria, and this is further combined with misdiagnosis and omission by the physician, which makes patients miss the correct treatment plan and timely treatment, posing a considerable challenge to both laboratory staff and clinicians. Providing accurate and prompt feedback from laboratories to clinicians will allow patients to receive the correct treatment plan as soon as possible. Furthermore, traditional biochemical identification is slow and less accurate for rare bacteria. Mass spectrometry can quickly and accurately identify rare bacteria; therefore, microbiology laboratories should be equipped with mass spectrometers (14), which represent an inevitable trend for the future development of clinical microbiology laboratories.

## Data availability statement

The original contributions presented in the study are publicly available. This data can be found here: http://www.ncbi.nlm.nih.gov/bioproject/857424 / BioProject ID: PRJNA857424.

## Ethics statement

This study was approved by the Chongqing University Jiangjin Hospital institutional review board. Written informed consent was obtained from the participants for the publication of the case report.

## Patient perspective

The patient agreed to our treatment plan and thanked us for the subsequent improvement in symptoms.

## Author contributions

YJ wrote and revised this manuscript. YZ, WQ, and JL reviewed this manuscript. YG and XL collected the data for this manuscript. All authors contributed to the article and approved the submitted version.

## Funding

This research was supported by the Key Technology Innovation Special of Key Industries of Chongqing Science and Technology Bureau Fund (cstc2020jscx-fyzxX0021).

## Conflict of interest

The authors declare that the research was conducted in the absence of any commercial or financial relationships that could be construed as a potential conflict of interest.

## Publisher's note

All claims expressed in this article are solely those of the authors and do not necessarily represent those of their affiliated organizations, or those of the publisher, the editors and the reviewers. Any product that may be evaluated in this article, or claim that may be made by its manufacturer, is not guaranteed or endorsed by the publisher.

## Abbreviations

WBC: white blood cell
CRP: C-reactive protein
PCT: procalcitonin
IL-6: interleukin 6
MIC: minimum inhibitory concentration.
